# Effect of the inclusion of mobile phone interviews to Vigitel

**DOI:** 10.1590/S1518-8787.2017051000171

**Published:** 2017-06-01

**Authors:** Regina Tomie Ivata Bernal, Deborah Carvalho Malta, Rafael Moreira Claro, Carlos Augusto Monteiro

**Affiliations:** I Núcleo de Pesquisas Epidemiológicas em Nutrição e Saúde. Faculdade de Saúde Pública. Universidade de São Paulo. São Paulo, SP, Brasil; II Departamento de Enfermagem Materno Infantil e Saúde Pública. Escola de Enfermagem. Universidade Federal de Minas Gerais. Belo Horizonte, MG, Brasil

**Keywords:** Interviews as Topic, Cell Phones, utilization, Frames, Data Collection, Health Surveys, Chronic Disease, epidemiology, Entrevistas como Assunto, Telefones Celulares, utilização, Sistema de Registros, Coleta de Dados, Inquéritos Epidemiológicos, Doença Crônica, epidemiologia

## Abstract

**OBJECTIVE:**

To evaluate the impact on the prevalence changes of risk factors for chronic diseases, published in the Surveillance System of Risk and Protection Factors for Chronic Diseases by Telephone Survey (Vigitel), after the inclusion of data from the population only with mobile phone.

**METHODS:**

Our study used data from the 26 State capitals and Federal District of Brazil obtained by the National Survey on Health (PNS) and Vigitel, both held in 2013. In each capital, we added a subsample of 200 adults living in households with only mobile phones, extracted from PNS, to the Vigitel 2013 database, with approximately 1,900 households, named Vigitel dual frame.

**RESULTS:**

Vigitel results showed absolute relative biases between 0.18% and 14.85%. The system underestimated the frequency of adult smokers (10.77%), whole milk consumption (52.82%), and soft drink consumption (22.22%). Additionally, it overestimated the prevalence of hypertension (25.46%). In the simulations using Vigitel dual frame, with inclusion of the sample of adults living in households with only mobile phones, the bias of estimates was reduced in five out of eight analyzed indicators, with greater effects in regions with lower rates of landline coverage. In comparing regions, we observed negative correlation (ρ = −0.91) between the percentage of indicators with presence of bias and the percentage of households with only mobile phone.

**CONCLUSIONS:**

The results of this study indicate the benefits of including a subsample of 200 adults with only mobile phone on the Vigitel sample, especially in the capitals of the North and Northeast regions.

## RESUMO

**OBJETIVO:**

Avaliar o impacto nas mudanças das prevalências de fatores de risco de doenças crônicas, divulgadas no Vigitel, após a inclusão de dados provenientes da população com somente telefone celular.

**MÉTODOS:**

O estudo utilizou os dados das capitais obtidos da Pesquisa Nacional de Saúde e do Vigitel, que foram realizados em 2013. Em cada capital, acrescentou-se uma subamostra de 200 adultos residentes em domicílios com somente celular, extraída da PNS, à base de dados do Vigitel 2013, com aproximadamente 1.900 domicílios, denominado Vigitel cadastro duplo.

**RESULTADOS:**

Os resultados do Vigitel mostraram vícios relativos absolutos entre 0,18% e 14,85%. O sistema subestimou a frequência de adultos fumantes (10,77%), o consumo de leite com teor integral de gordura (52,82%) e o consumo de refrigerante (22,22%). Adicionalmente, superestimou a prevalência de hipertensão (25,46%). Nas simulações utilizando o Vigitel cadastro duplo, com inclusão da amostra de adultos residentes em domicílios com somente celular, o vício das estimativas foi reduzido em cinco de oito indicadores analisados, com maiores efeitos nas regiões com menores taxas de cobertura de telefonia fixa. Na comparação entre as regiões, observa-se correlação negativa (ρ = -0,91) entre o percentual de indicadores com presença de vício e o percentual de cobertura de domicílios com somente celular.

**CONCLUSÕES:**

Os resultados do presente estudo indicam os benefícios da inclusão de uma subamostra de 200 adultos com somente celular na amostra do Vigitel, especialmente nas capitais das regiões Norte e Nordeste.

## INTRODUCTION

In 2015, the population-based Surveillance System of Risk and Protection Factors for Chronic Diseases by Telephone Survey (Vigitel), conducted in the 26 capitals and Federal District of Brazil, completed 10 years^1^. The advantages of adopting a surveillance system by telephone are numerous: practicality; agility to detect changes in trends; lower cost; quickness in collecting information; besides the opportunity to continually support the planning of public policies and guidelines of programs for health promotion and prevention of risk and chronic noncommunicable diseases (CNCD)^2^.

Vigitel uses, since its origin, post-stratification weights to correct the bias resulting from the exclusion of the population segment without landline. This strategy aims to equate the sample studied by Vigitel (population with landline) with that of the cities studied (with and without landline) according to predefined features^1^. From 2006 to 2011, the system used population data from Census 2000, by the Brazilian Institute of Geography and Statistics (IBGE)^a^, to construct post-stratification weights by the cell weighting method^3^. During this period, several studies evaluated the presence of biases in estimates published by Vigitel, by comparing results obtained in household surveys and Vigitel. The results presented biases in some indicators released by Vigitel, in cities with high^4-7^, average^4,8^, and low^4,9,10^ landline coverage. In Rio Branco, because of the exclusion of 60% of households without access to landline, Bernal et al.^9^ showed that the post-stratification weights of Vigitel did not fix potential biases of the prevalence of practice of physical activity in free time, hypertension, asthma, asthmatic bronchitis, chronic bronchitis, or emphysema in the city.

In 2012, Vigitel changed the methodology of constructing post-stratification weights from cell to rake^11^. The rake method allows the use of different sources of population data, even in the intercensal period, for estimating the post-stratification weights. For each capital, annual population estimates were obtained by age group (six categories) and by education level stratified by sex (eight categories). This method works one variable at a time, equating the total distribution of the variable in the sample, weighted by the sample weights, and in the population, by iteration procedures. This process is then repeated in each variable used in the construction of weights, causing the sample distribution to be identical to that of the population for these variables. The new post-stratification weights of Vigitel, for each capital, were estimated in the SAS statistical package using the rakinge.sas macro^12^. Thus, Vigitel results follow the demographic transition of the population^13^.

However, these adjustments are not sufficient to eliminate biases arising from the insufficient landline coverage. Data from Census 2010^b^ show that 61% of private households located in the 26 capitals and Federal District have landline, with heterogeneous distribution in the Country. The North and Northeast regions present 38% and 44% coverage, respectively, while the Midwest, South, and Southeast regions have 56%, 70%, and 74% coverage, respectively. These data show that the frame of landline subscribers in the North region excludes at least 62% of the study population. The Northeast and Midwest regions have exclusion of at least 56% and 44%, respectively. This scenario of low landline coverage can lead to biased estimates, being especially relevant in the capitals of North, Northeast, and Midwest regions. In the South and Southeast, with higher coverage rates, we expect the introduced biases to be negligible.

However, because of the technological changes that have occurred in recent years, the monitoring of the coverage of landline and mobile telephony in the capitals is essential to Vigitel. In 2013, the Annual Report of Anatel^c^ showed exponential increase of the access to personal mobile service since the early 2000s, with slowing growth since 2012.

Also, data from the National Survey on Health 2013^d^ indicate that the Southeast, South, and Midwest regions present the largest landline coverage, ranging between 61% and 75%, while the North and Northeast present coverage of 34% and 44%, respectively. However, these regions with low landline coverage have a high coverage of households with only mobile phones (63% in the North and 54% in the Northeast)^d^.

This study aimed to assess the impact of including data from the population with only mobile phones in the Vigitel estimates.

## METHODS

Our study used data from the National Survey on Health (PNS)^d^ and Vigitel^14^, both held in 2013.

PNS is population-based household survey conducted by IBGE in partnership with the Brazilian Ministry of Health, with 62,986 households interviewed across the Country. The sampling procedure was composed of simple random sampling, by conglomerates, divided into three stages: census tracts (primary units); households (secondary units); and an adult dweller – 18 years or older – (tertiary unit), selected from the list of residents built at the moment of interview to respond to the specific questionnaire. Were defined sample weights for primary sampling units, households and all their residents, and the weight for the selected dweller. The minimum size of the sample was 1,800 households per Federative Unit^15^. This study used a sample of adults aged 18 years or older living in residence without landline, in the urban areas of the 26 State capitals and Federal District, totaling 28,493 interviews.

Vigitel, in turn, is a telephone survey conducted by the Brazilian Ministry of Health in partnership with the Center for Epidemiological Research in Nutrition and Health of USP (NUPENS/USP) since 2006. The target population are adults (≥ 18 years) living in the 26 capitals of Brazil and in the Federal District. The system uses the electronic registers of landlines to randomly select probabilistic samples of residential lines. Initially, they randomly select 5,000 landlines in each city. These samples are divided into 25 replicas with size equal to 200 in each one, using the same process of random selection of the initial sample. From the identification of landlines eligible to the study (residential and active lines), one adult living in the house is randomly selected to be interviewed. In 2013, year of the Vigitel edition used in this study, the interviews were conducted from February to December, with minimum sample size of 1,900 interviews per city, reaching a total sample of 52,929 respondents. Post-stratification weights are used to adjust the sample distribution (with landline), according to age, sex, and education level, for the total population (with and without landline)^14^.

More information about the sampling process of both surveys can be obtained in the original publication of their results^14,15^.

### Landline and Mobile Phone Coverage

PNS surveyed the ownership, by households, of landline and mobile phone. These data were used in the first step to describe landline coverage by region and, also, the sociodemographic profile of the population according to age, education level, and sex, stratified by telephone ownership profile (according to three categories: landline, mobile phone only, and no phone) and by region.

### Estimate of Bias in Vigitel Estimates

The second stage of the study consisted of estimating the Vigitel bias by comparing the frequencies obtained in Vigitel and PNS. In this stage, we selected the indicators considered comparable (from the questions and response options that are similar between both surveys). They are: frequency of adult smokers; consumption of red meat or chicken with excess fat; whole milk consumption; regular (≥ 5 days/week) soft drink, candy, and bean consumption; and self-reported medical diagnosis of hypertension and diabetes. We adopted the frequency estimated in PNS as gold standard in estimating the absolute relative bias expressed by: (1) $\frac{\left|\%Vigitel\;-\%PNS\right|}{\%PNS}$. Both PNS and Vigitel data were weighted to adjust the sociodemographic distribution of the sample of these surveys to that used as a reference in PNS, which uses the population estimate produced by the Coordination of Population and Social Indicators. The test of Means from two populations was used as a criterion of bias detection with a 5% significance level.

### Estimate of the Impact of Including Interviews by Mobile Phone to Vigitel

The third step of the study consisted in the simulation of dual frame, composed of landline and mobile phone samples in Vigitel 2013. Following Kish’s^15^ proposal, in each capital, we added a subsample of 200 adults living in households only with mobile phones to the Vigitel 2013 sample, with approximately 1,900 households, named Vigitel dual frame. This subsample was extracted from the PNS by systematic sampling, with age, education level, and sex as control variables, maintaining the distribution of the population living with only mobile phones in each capital. We adopted the frequency estimated in PNS as gold standard in estimating the absolute relative bias expressed by: (2) $\frac{\left|\%Vigitel\;dual\;frame\;-\%PNS\right|}{\%PNS}$. For analyzing Vigitel dual frame data – landline and mobile phone –, we used the rake method to build post-stratification weights in each capital, considering the sample weight (adults/number of phones) of Vigitel and V0029 (weight of selected resident with non-interview correction without calibration by the projection of population for selected resident) of the mobile phone subsample of PNS as their respective design weights, and the population estimated by PNS as external source for estimating the post-stratification weights of the dual frame.

The impact of including the subsample of adults living in households with only mobile phone on the Vigitel sample was defined by the expression: (3) $\frac{Bias\;\left(\left[\left(Vigit\;dual\;frame\right)\;-\;Bias\left(Vigitel\right)\right]\right)}{Bias\left(Vigitel\right)}\times 100$.

PNS was approved by the National Research Ethics Committee (Process: 328,159, June 26, 2013). Vigitel was approved by the National Human Subject Research Ethics Committee (Processes 13081/2008 and 355,590/2013). All individuals were consulted, the surveys were clarified to them, and they agreed to participate.

## RESULTS

### Telephone Coverage and Population Profile

**Figure 1:**
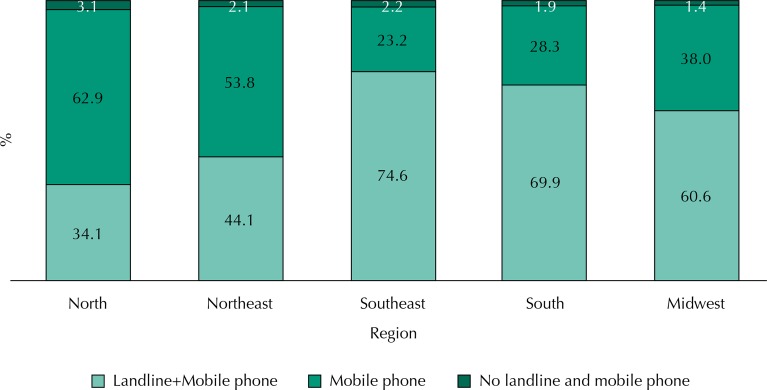
Distribution of residential landline coverage, according to set of capitals of each macro-region of the Country. PNS, 2013*.

The Southeast, South, and Midwest regions presented the largest landline coverage, ranging between 61% and 75%, while the North and Northeast presented coverage of 34% and 44%, respectively. The highest frequencies of households with only mobile phone were observed in the capitals of the North and Northeast regions, with 62.9% and 53.8%, respectively, with low landline coverage. The Midwest, South, and Southeast presented 38.0%, 28.0%, and 23.2%, respectively. The proportion of households without any telephone tended to be small, ranging from 1.4% in all the capitals of the Midwest to 3.1% in the capitals of the North (Figure 1).

* Interviews held with adults aged 18 years or older in the urban areas of the 26 State capitals and in the Federal District (n = 28,493). For more information, see Methods.

**Figure 2:**
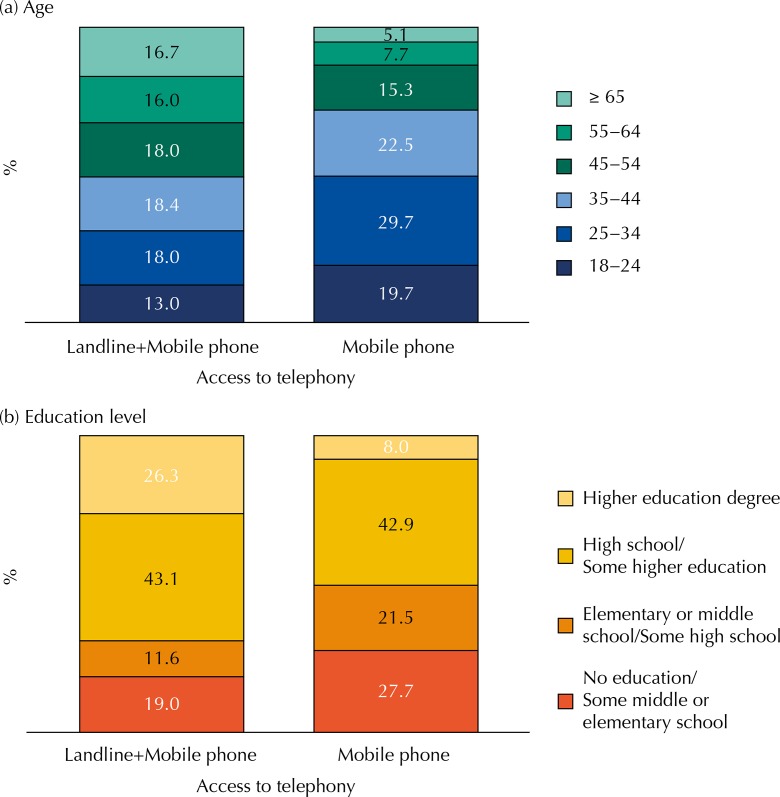
Distribution by age and educational level (%) according to type of access to telephony stratified by geographic region. Adult population living in the capitals, 2013.

The sociodemographic profile of the population living in household with only mobile phone differs from the population with landline in all regions, the main difference being the higher frequency of individuals in lower age groups, with less than 34 years old, and with education level up to some high school (Figure 2). In all regions, the proportion of men or women living in households with only mobile phone is similar to the proportion of men or women living in households with landline.

### Bias Estimates

In the evaluation of bias estimates of Vigitel, adopting the results of PNS as gold standard, one can observe non-negligible biases in the frequency of adult smokers, in whole milk, soft drink, and candy consumption, and in the prevalence of hypertension and diabetes. A similar result was observed between men and women, except for prevalence of diabetes (Table 1). These biases varied between geographic regions (Table 2). In the North, Vigitel underestimates the frequency of adult smokers and soft drink consumption and overestimates the frequency of consumption of red meat or chicken with fat and of candy and the prevalence of hypertension and diabetes. In the Northeast, Vigitel underestimates the frequency of adult smokers and whole milk, soft drink, and bean consumption and overestimates the consumption of red meat or chicken with fat and the prevalence of hypertension. In the Southeast, Vigitel underestimates the whole milk and candy consumption and overestimates the prevalence of hypertension. In the South and Midwest, Vigitel underestimates the whole milk, soft drink, and candy consumption and overestimates the prevalence of hypertension (Table 2).

**Table 1:**
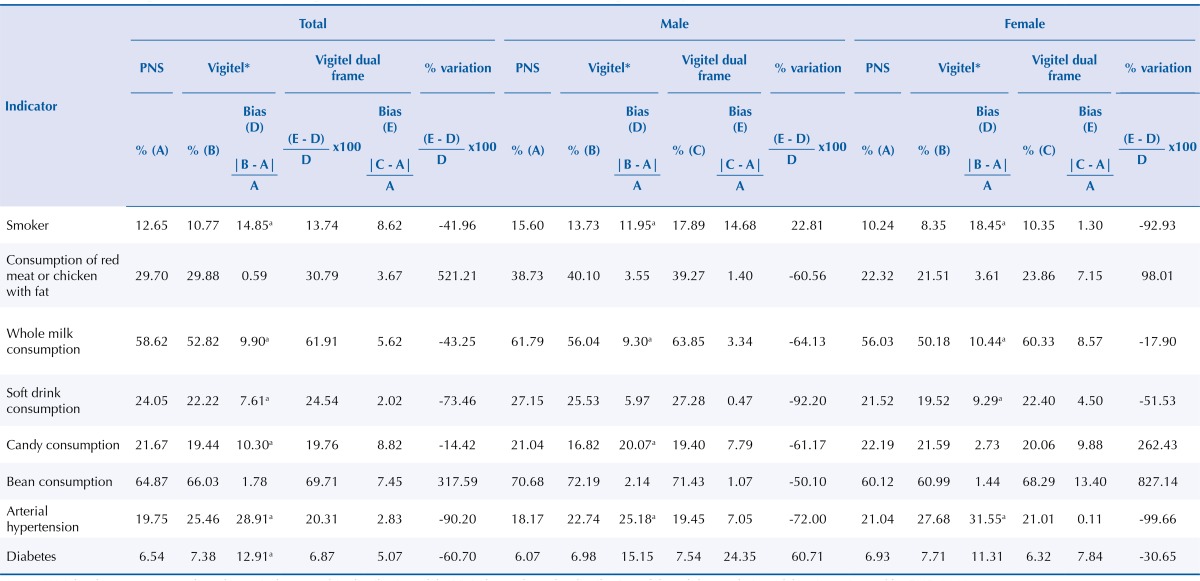
Estimates of Vigitel 2013 biases according to indicators by sex. Population of adults living in the capitals, 2013.

Note: * Weighted percentage to adjust the sociodemographic distribution of the Vigitel sample to the distribution of the adult population of the city estimated by PNS, 2013.

Vigitel: Surveillance System of Risk and Protection Factors for Chronic Diseases by Telephone Survey.

^a^ non-negligible bias (p-value < 5%).

**Table 2:**
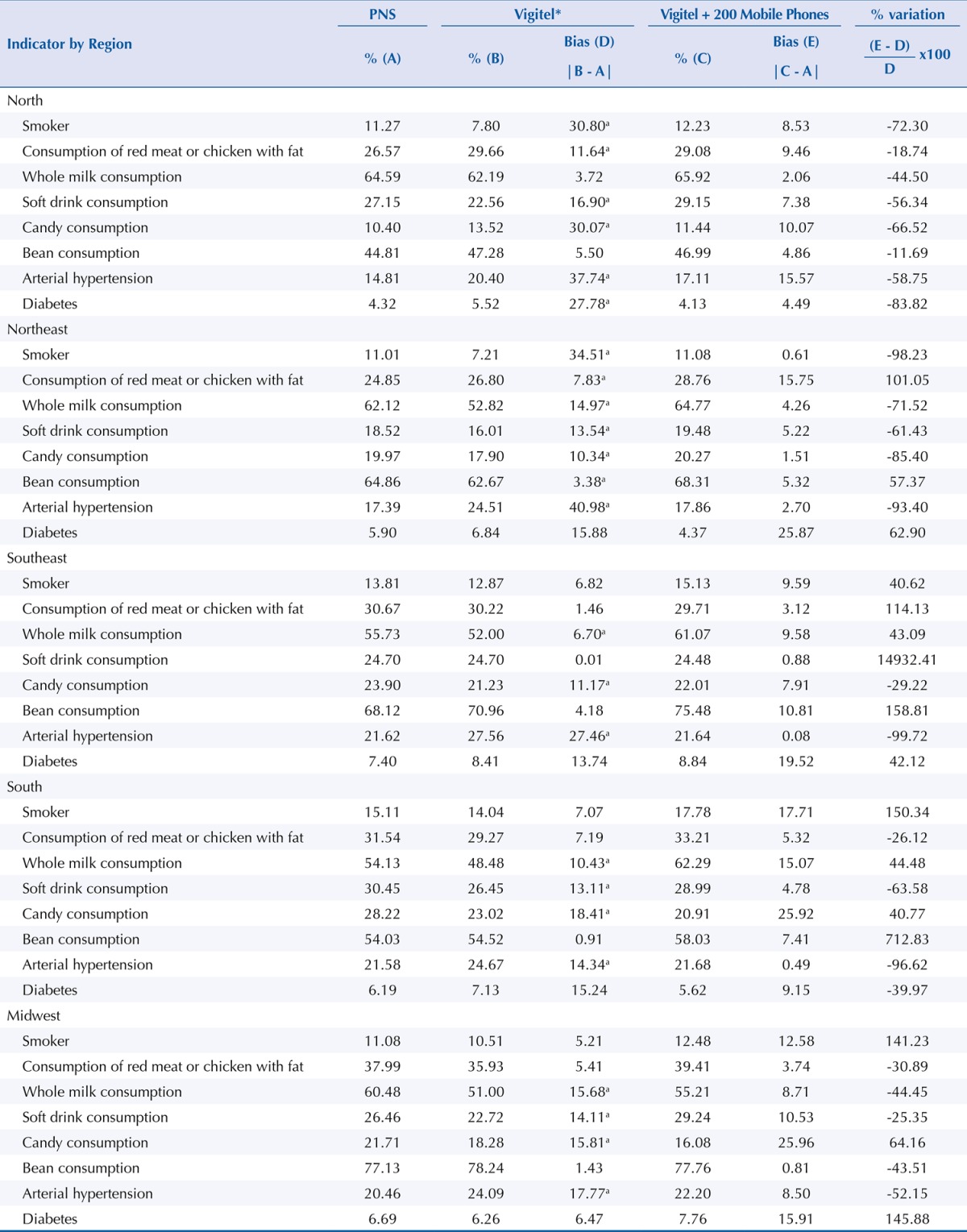
Estimates of Vigitel 2013 biases according to indicators by region and sex. Population of adults living in the capitals, 2013.

Note: * Weighted percentage to adjust the sociodemographic distribution of the Vigitel sample to the distribution of the adult population of the city estimated by PNS, 2013.

Vigitel: Surveillance System of Risk and Protection Factors for Chronic Diseases by Telephone Survey.

^a^ non-negligible bias (p-value < 5%).

The use of dual frame intensely reduced the biased estimates of Vigitel, especially in the capitals of regions with lower landline coverage. In the North, we observed bias reduction in the eight indicators studied, ranging from -11.7% in the case of regular consumption of beans to -83.8% in the case of medical diagnosis of diabetes. We observed a similar scenario in the Northeast and Midwest, where bias was reduced in five out of eight indicators studied. On the other hand, in the South and Southeast, bias was reduced in three and two of the indicators, respectively, and increased in other indicators (Table 2).

In comparing regions, we observed negative correlation (ρ = -0.91) between the percentage of indicators with presence of bias and the percentage of households with only mobile phone, i.e., increased bias as the percentage of houses with only mobile phone decreases.

## DISCUSSION

PNS 2013 provided an important evaluation regarding methodological changes required for the effective continuity of Vigitel. Data from PNS 2013 indicate that the capitals of the Southeast, South, and Midwest have the higher landline coverage, ranging between 61% and 75%, while the North and Northeast present coverage of 34% and 44%, respectively. Places with low landline coverage present the highest proportions of households with only mobile phone, and this was the modal condition in all the capitals of North (63%) and Northeast (54%).

In the evaluation of the estimates of relative absolute biases of Vigitel, the results show biases with the current methodology of the system. The system underestimates the percentage of adult smokers (10.77%), whole milk consumption (52.82%), soft drink consumption (22.22%), and candy consumption (19.44%) and overestimates the prevalence of hypertension (25.46%). The biggest biases were observed in the capitals of the regions with lower landline coverage: North, Northeast, and Midwest.

To completely eliminate the biases of the estimates disclosed in the telephone survey, Groves et al.^16^ and Brick and Lepkowski^17^ recommend the use of alternative methods, such as dual-frame and multiple mode and frame surveys. These methods take more than one register for random selection and present advantages regarding the current telephone survey. However, the major challenge consists of the statistical processing to obtain valid estimates for the data from multiple frames. Currently, several telephone surveys use weighting methods to obtain estimates of dual frame^18,19^. With the advancement of technology, recently, algorithms for this type of approach are already available in several statistical packages. In the R program, the frames^20^ package, for example, offers eight functions, with different estimators, for estimating mean and total, and also offers the compare function, which describes the summary measures of the results to help choosing the best estimator for obtaining valid estimates.

A similar scenario to that observed in Brazil, of increased frequency of households with only mobile phone, has already been observed in developed countries, such as USA^21^ and Australia^22^, requiring the inclusion of new frame (or modes of data collection) in health surveys traditionally conducted by telephone interviews^19,21,22^.

Since 2011, the Behavior Risk Factor Surveillance System (BRFSS)^23^, in the United States, uses telephone and mobile phone surveys for including adults living in households with only mobile phone. According to data from the National Health Interview Survey (NHIS), in the first semester of 2014, 45.4% of households had only mobile phone. The system adopts the random digit dialing method for obtaining samples of landline, ranging between 1,842 and 12,962 interviews, while mobile phone samples come from commercial register, varying between 868 and 8,674 interviews. In the statistical analysis of data, BRFSS adds the database of the mobile phone survey to the database of the landline survey to obtain estimates of indicators. The system adopts the rake weighting method for obtaining valid estimates.

It is worth mentioning that the process of change in the BRFSS methodology began in 2009 with pilot studies using samples of landlines and mobile phones^24,25^. During this period, several specialists have recommended weighting method, by the dissemination of studies that evaluated the dual frame and the different variables used in the construction of post-stratification weights, to reduce bias due to low response rate^26,27^.

As well as BRFSS, the New South Wales Population Health Survey, conducted in Australia, added the mobile phone to the telephone survey in 2012, after several pilot studies^28^. In 2012, the survey used 1,224 (36.1%) mobile phones and 2,171 (63.9%) landlines, at a cost of $74.42 and $31.13 per interview, respectively. The results show that the inclusion of mobile phone did not affect the response rates nor the disclosed estimates; however, it included the young population and the indigenous people living in Torres Strait Islands, Australia, thus expanding the register coverage^28^.

Brazil follows this trend. In 2008, the Health Surveillance Secretariat carried out the first pilot study of Vigitel using mobile phone survey in Belo Horizonte, with high landline coverage, and in Maceio, with low landline coverage. At the time, both individuals who had only mobile phone and those who lived in houses with landline were interviewed. In the comparison between estimates obtained in the population with landline and mobile phone, the results did not indicate the need for inclusion of mobile phone interviews to the traditional operation of Vigitel (the results showed no difference between estimates)^29^.

However, the increase in number of households with only mobile phone required the inclusion of this strategy to be still considered. The simulations performed in this experiment (Vigitel dual frame) propose a new approach for including individuals interviewed exclusively by mobile phone to Vigitel (inclusion of about 200 individuals living in households without a landline in the regular sample of Vigitel) and its results attest its effectiveness by showing the intense bias reduction in the frequency of the indicators analyzed, especially in the capitals of regions with lower residential landline coverage. However, the inclusion of this sample in the capitals with high landline coverage has increased bias in six indicators in the Southeast region and four in the South. In the Midwest, with average landline coverage, the bias increased in three out of eight indicators.

Between regions, the results of the Vigitel dual frame simulation show bias reduction in all indicators of the North region, which has coverage of houses with only mobile phone of about 63%. In the Northeast, with 54% coverage of houses with only mobile phones, a bias reduction can be observed in five out of eight indicators. The Midwest, with 38% coverage, presented bias reduction in five of the eight indicators (63%). The South, with 28% coverage, presented reduction in three out of eight indicators (38%) and the Southeast, with 23% coverage, reduction in two out of eight indicators (25%). Thus, in the Southeast, South, and Midwest, the current Vigitel offers better performance when compared to Vigitel dual frame.

The main limitation of this study is connecting data obtained in the household and telephone surveys, with differences that can be subjected to interference from the methodological effect of the survey, sampling type, questions, means of data collection, interview time, and duration of the survey.

The proposal of including mobile phones in Vigitel using random digit dialing method requires great effort, with many calls to identify the adult population living in the capital, which increases the research cost. In the first pilot study of Vigitel, held in 2008 using the mobile phone survey in Belo Horizonte, with high landline coverage, and in Maceio, with lower landline coverage, the cost was 6.6 times greater than the cost of the interview conducted by landline, value much higher than that found in the United States (2.4 times) (including financial incentive to the respondent)^29^.

The proposal of including a small sample of adults living in households with only mobile phones to the Vigitel sample aims to reduce the number of calls to find the target population of the survey, comprised of adults living in the capitals. On the other hand, this sample needs to be controlled by age, education level, and sex, i.e., this is a quota sampling. Thus, there will be inclusion of the population excluded by Vigitel, mostly composed by young people with elementary and high school.

This proposal differs from BRFSS and New South Wales Population Health Survey, with mobile phone samples corresponding to almost half the size of the landline samples. This strategy is useful because it does not need to use quota sampling, but, on the other hand, the survey cost increases.

## CONCLUSION

In the simulation of Vigitel dual frame, the results show bias reduction in five out of eight indicators analyzed. In comparing regions, we observed negative correlation (ρ = -0.91) between the percentage of indicators with presence of bias and the percentage of households with only mobile phone, i.e., the bias increases as the percentage of houses with only mobile phone decreases. In the North and Northeast, Vigitel dual frame presents good performance in reducing Vigitel bias, while in the Southeast, South, and Midwest, the current Vigitel offers better performance when compared to Vigitel dual frame.

Therefore, the inclusion in Vigitel of a subsample of 200 adults with only mobile phone reduced, in the North and Northeast, the bias of samples of landlines registers in Brazil. We recommend further Vigitel pilot studies, interviewing users who have only mobile phones, to compare with the findings presented here.

## Footnotes

a Instituto Brasileiro de Geografia e Estatística. Censo demográfico 2000: downloads: estatísticas. Rio de Janeiro: IBGE; s.d. [cited 2013 Dec 12]. Available from: http://downloads.ibge.gov.br/downloads_estatisticas.htm

b Instituto Brasileiro de Geografia e Estatística. Microdados do Censo 2010: downloads: estatísticas. Rio de Janeiro: IBGE; s.d. [cited 2013 May 14]. Available from: http://downloads.ibge.gov.br/downloads_estatisticas.htm

c Agência Nacional de Telecomunicações. Relatório anual 2013. Brasília (DF): ANATEL; 2013 [cited 2016 May 13]. Available from: http://www.anatel.gov.br/Portal/verificaDocumentos/documento.asp?numeroPublicacao=312603&pub=original&filtro=1&documentoPath=312603.pdf

d Instituto Brasileiro de Geografia e Estatística. Pesquisa Nacional de Saúde 2013: microdados. Rio de Janeiro: IBGE; 2013 [cited 2016 Jun 27]. Available from: http://www.ibge.gov.br/home/estatistica/populacao/pns/2013/default_microdados.shtm
